# Lead in Drinking Water as a Public Health Challenge

**DOI:** 10.1289/ehp.1001979

**Published:** 2010-04

**Authors:** Björn P. Zietz, Jessica Laß, Roland Suchenwirth, Hartmut Dunkelberg

**Affiliations:** Governmental Institute of Public Health of Lower Saxony (Niedersächsisches Landesgesundheitsamt), Hannover, Germany, E-mail: bjoern.zietz@nlga.niedersachsen.de; Medical Institute of General Hygiene and Environmental Health, University of Göttingen, Göttingen, Germany

In drinking water supplies the intake of the toxic heavy metal lead is commonly due to metal corrosion in the peripheral water distribution system, especially the user’s plumbing or lead service lines. Recently, the problem again received attention in the United States when testing data of drinking water at schools was published (Renner 2009). In Europe several countries are known to have significant numbers of buildings with elevated lead tap water concentrations, for example, the United Kingdom (Watt et al. 1996), Austria (Haider et al. 2002) and Germany (Becker et al. 2001).

Lead exposure from drinking water has been a topic of public health prevention programs in several parts of Germany before, for example, Hamburg (Fertmann et al. 2004) and Frankfort (Hentschel et al. 1999). In 2005 in the northern German state of Lower Saxony, a prevention program was initiated comprising three different approaches at the same time to achieve a widespread effect. To assess the present state of drinking water contamination with lead, a free examination of lead in tap water (after nocturnal stagnation) was offered in cooperation with local public health departments for private households that included young women and families with children (Zietz et al. 2007, 2009). Along with the collection of data, the program aimed to focus public attention on this public health problem. In another part of this program, data from local public health departments on existing lead measurements, especially in public buildings, were collected and analyzed (Zietz et al. 2007, 2009). Finally, a working group on lead replacement, consisting of representatives of all relevant parties (e.g., tenant and landlord associations, crafts people, building and health administrations) was initiated. In the screening part of the project, a total of 2,901 tap water samples from households were collected during 2005–2007. Of these, 7.5% had lead concentrations > 10 μg/L (recommended limit of the World Health Organization) and 3.3% had concentrations above the present limit of the German drinking water ordinance (25 μg/L) (Zietz et al. 2009). We found remarkable regional differences in the frequency of tap water contamination. An additional inclusion criterion in this study was that buildings must have been constructed before 1974 (after which no new lead pipes were installed); therefore, the results cannot be compared directly to other studies. From the data, we roughly estimated that about 4.7% of all households in Lower Saxony have lead concentrations > 10 μg/L (Zietz et al. 2009). In an earlier study in southern Lower Saxony (Zietz et al. 2001a), households with mothers of newborn babies from the area around the university city of Göttingen were investigated. Of the 1,434 stagnation samples, 3.1% had lead concentrations > 10 μg/L.

A moderately higher percentage of households with elevated composite water samples was found in the geographic area of the city of Berlin using two composite water sampling methods (5.6% and 7.0%, respectively. In total, 2,109 households were tested with both methods in the federal state of Berlin (Zietz et al. 2001b). In a representative study of samples collected in all parts of Germany during 1997–1999 (Becker et al. 2001), the 90th percentile of lead concentrations in 4,761 stagnation samples was 7.6 μg/L.

Projects in association with epidemiologic investigations also provide an opportunity to design prevention programs in this field. Generally, we favor the precautionary measure of preventing exposure to lead by replacing pipes completely. The addition of anticorrosive substances to the public water supply can be effective in lowering lead concentrations. In contrast, changing water chemistry (e.g., a new water disinfectant method, as in Washington, DC, USA) can have a substantial effect in elevating lead (Renner 2009). Flushing the water pipes and using only cold water are short-term methods of decreasing exposure to lead from tap water. Using bench-top water filters can also decrease lead concentrations, but problems such as leaching of different substances into the water or microbial contamination may arise under certain conditions. Thus, lead plumbing material in buildings still poses a challenge for public health in the United States and in Europe.
